# Outcomes of the SpineJack Implant System for Cancer Patients With Pathological Fractures

**DOI:** 10.1111/papr.70094

**Published:** 2025-10-16

**Authors:** Alaa Abd‐Elsayed, Christopher Gilligan

**Affiliations:** ^1^ Department of Anesthesiology University of Wisconsin School of Medicine and Public Health Madison Wisconsin USA; ^2^ Robert Wood Johnson University Hospital New Brunswick New Jersey USA

The SpineJack system is an FDA‐approved therapy for vertebral compression fractures (VCFs) with efficacy comparable to balloon kyphoplasty.

Parmar et al. [1] conducted a retrospective study to examine the efficacy and safety of the SpineJack system. The authors collected data from 67 cancer patients who received 94 SpineJack procedures.

The authors found a significant reduction in pain scores and a reduction in opioid use in patients who received the SpineJack implant.

The authors concluded that the use of the SpineJack system for VCFs is effective and safe.

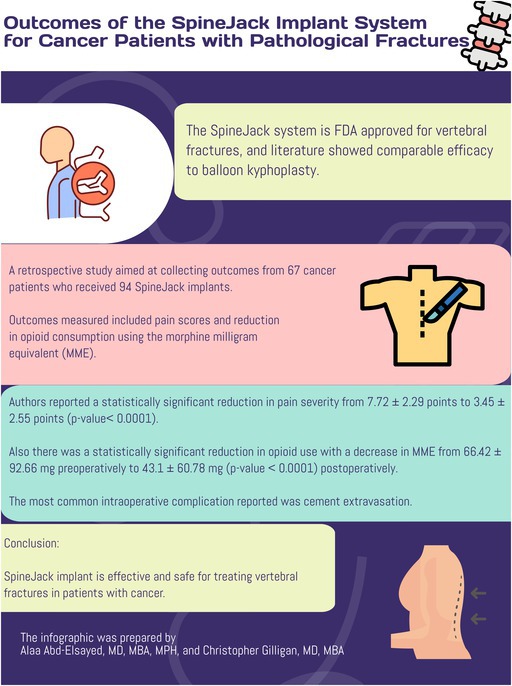

## Conflicts of Interest

Dr. Christopher Gilligan is the editor in chief of pain practice, and Dr. Alaa Abd‐Elsayed is a section editor of pain practice.

## Data Availability

Data sharing is not applicable to this article as no datasets were generated or analyzed during the current study.
